# High incidence of imperforate vagina in ADGRA3-deficient mice

**DOI:** 10.1186/s12915-024-01873-6

**Published:** 2024-04-08

**Authors:** Jone Marita Kvam, Maja Lind Nybo, Lola Torz, Riia Karolina Sustarsic, Kristian Høj Reveles Jensen, John Erik Nielsen, Hanne Frederiksen, Sarina Gadgaard, Katja Spiess, Steen Seier Poulsen, Jesper Skovhus Thomsen, Pamela Cowin, Martin Blomberg Jensen, Takeshi Kurita, Mette Marie Rosenkilde

**Affiliations:** 1 Department of Biomedical Sciences, Faculty of Health and Medical Sciences, University of Copenhagen, Copenhagen, Denmark; 2 Department of Growth and Reproduction and International Center for Research and Research Training in Endocrine Disruption of Male Reproduction and Child Health (EDMaRC), Copenhagen University Hospital - Rigshospitalet, Copenhagen, Denmark; 3 Bainan Biotech, Copenhagen, Denmark; 4 Department of Biomedicine, Aarhus University, Aarhus, Denmark; 5 Departments of Cell Biology and Dermatology, New York University School of Medicine, New York, NY USA; 6 Group of Skeletal, Mineral and Gonadal Endocrinology, Department of Growth and Reproduction, Rigshospitalet, University of Copenhagen, Copenhagen, Denmark; 7 Division of Translational Endocrinology, Department of Endocrinology and Internal Medicine, Copenhagen University Hospital – Herlev and Gentofte, Copenhagen, Denmark; 8 Department of Cancer Biology and Genetics, Ohio State University, Columbus, OH USA

**Keywords:** Adhesion GPCR, ADGRA3, Female fertility, Imperforate vagina

## Abstract

**Background:**

Ten percent of the female population suffers from congenital abnormalities of the vagina, uterus, or oviducts, with severe consequences for reproductive and psychological health. Yet, the underlying causes of most of these malformations remain largely unknown. ADGRA3 (GPR125) is involved in WNT signaling and planar cell polarity, mechanisms vital to female reproductive tract development. Although ADGRA3 is a well-established spermatogonial stem cell marker, its role within the female urogenital system remains unclear.

**Results:**

In this study, we found *Adgra3* to be expressed throughout the murine female urogenital system, with higher expression pre-puberty than after sexual maturation. We generated a global *Adgra3*^*−/−*^ mouse line and observed imperforate vagina in 44% of *Adgra3*^*−/−*^ females, resulting in distension of the reproductive tract and infertility. Ovarian morphology, plasma estradiol, ovarian *Cyp19a1*, and vaginal estrogen receptor α (*Esr1*) expression were unaffected. However, compared to controls, a significantly lower bone mineral density was found in *Adgra3*^*−/−*^ mice. Whereas vaginal opening in mice is an estrogen-dependent process, 17β-estradiol treatment failed to induce vaginal canalization in *Adgra3*^*−/−*^ mice. Furthermore, a marked reduction in vaginal and ovarian progesterone receptor expression was observed concomitant with an upregulation of apoptotic regulators *Bcl2*, *Bid*, and *Bmf* in adult *Adgra3*^*−/−*^ females with a closed vagina.

**Conclusions:**

Our collective results shed new insights into the complex mechanisms by which the adhesion receptor ADGRA3 regulates distal vaginal tissue remodeling during vaginal canalization via altered sex hormone responsiveness and balance in apoptotic regulators. This highlights the potential of ADGRA3 as a target in diagnostic screening and/or therapy for obstructive vaginal malformations in humans.

**Supplementary Information:**

The online version contains supplementary material available at 10.1186/s12915-024-01873-6.

## Background

Normal development and function of the female reproductive tract are indispensable for mating, fertilization, implantation, embryonic development, and safe delivery of offspring. However, up to 10% of women suffer from congenital abnormalities of the vagina, uterus, or oviducts [1–3], which has devastating consequences for their reproductive and psychological health [4]. The most common congenital obstructive anomalies of the reproductive tract are imperforate hymen (observed in 1:1000 females), followed by a transverse vaginal septum and vaginal atresia [5, 6]. Despite occurring at high frequencies, the etiologies of most vaginal abnormalities are unknown [7]. An increased effort toward understanding basic biology and underlying causes of vaginal malformations is necessary to improve diagnosis and appropriate treatment.

Although the anatomy of mice differs slightly from that of humans, the signaling pathways are largely conserved, making mouse models an invaluable tool for investigating female reproductive tract development [8, 9]. The female reproductive tract originates from the mesodermal Müllerian ducts and is guided by the Wolffian ducts to grow caudally, eventually reaching the endodermal urogenital sinus [9]. Fusion of the Müllerian ducts results in the formation of the uterovaginal canal. The urogenital sinus subsequently forms the sinovaginal bulbs that develop into the solid vaginal plate after proliferation. Growth and canalization of the vaginal plate occur simultaneously to form the lower part of the proximal vagina and vaginal orifice [9]. In mice, this process occurs at puberty via estrogen-mediated induction of apoptosis of the distal vaginal epithelium to generate the vaginal entrance [10–12]. Thus, the opening of the murine vagina remains closed at birth and is sensitive to local and systemic alterations in estrogen signaling that accompany sexual maturation [12–14]. The exact apoptotic mechanism that produces the vaginal opening remains unclear, but Müllerian duct development depends on proper WNT signaling [9, 15]. In support of a complex mechanism of vaginal development, obstructive vaginal malformations and failure of opening have been reported in several mouse strains, including those with genetic mutations in components of the WNT pathway cascades [16–21]. They have also been reported in mice with mutations in tumor suppressors (LHFPL2^G102E^ and trBraca1 + p53^+/−^) [22, 23], overexpression of apoptotic regulator BCL2 [11], and mono, dual, or triple knockout of other genes involved in apoptosis, such as *Bax*, *Bak*, *Bmf* [24–26], and other genes involved in cellular signaling [27–35].

**Fig. 1:**
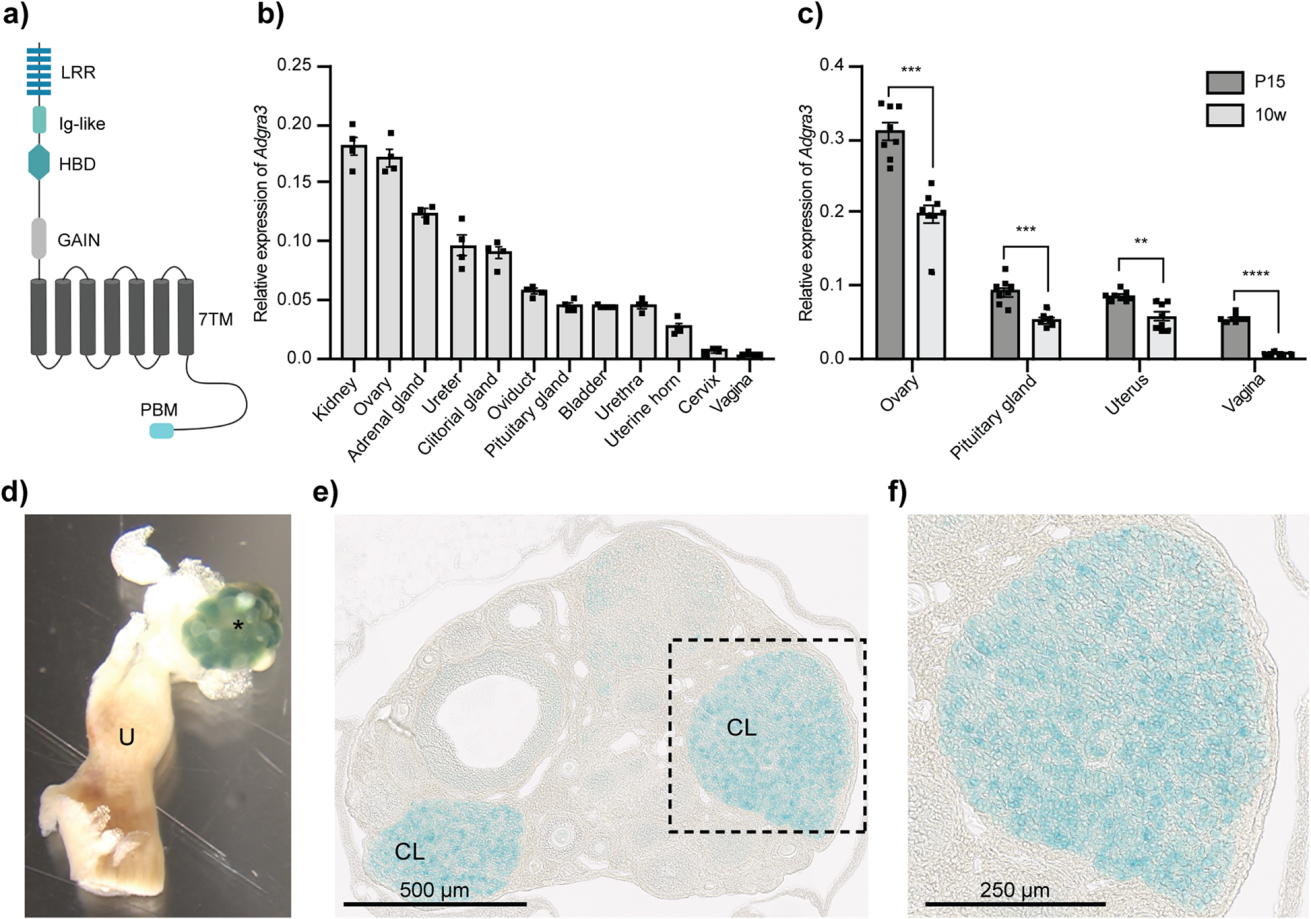
*Adgra3* is expressed during stages of vaginal remodeling and in the corpus luteum. **a** Illustrative structure of ADGRA3 (also known as GPR125). 7TM, seven transmembrane domain; GAIN, GPCR auto-proteolysis inducing domain; HBD, hormone binding domain; Ig-like, immunoglobulin-like; LRR, leucine-rich repeats; PBM, PDZ-binding motif. **b**, **c** Endogenous *Adgra3* expression in **b** whole tissues of the adult female urogenital system harvested from 8- to 10-week-old C57BL/6J females in proestrus (*n* = 4) and **c** in the ovary, pituitary gland, uterus, and vagina of premature (P15, *n* = 8) and mature (8–10 weeks, *n* = 8) *Adgra3*^+*/*+^ mice in proestrus. Relative expression was calculated relative to the mean expression of housekeeping genes *36b4* and *Ywhaz* using the 2^−∆CT^ method, and significance was determined by unpaired *t*-test (ovary: ***p = 0.0001; pituitary gland: ***p = 0.0003; uterus: **p = 0.0024; vagina: ****p < 0.0001). Each dot represents an individual animal. Data are presented as mean ± SEM. **d** ADGRA3-β-gal expression in whole ovarian mount from an adult *Adgra3*^*lz/*+^ mouse. The asterisk (*) indicates the ovary. U, uterine horn. **e**, **f** Representative images of a paraffin-embedded section of X-gal-stained ovarian whole mount from an *Adgra3*^*lz/*+^ mouse (*n* = 3), with expression located in the granulosa lutein cells within the corpus luteum (**f**). CL, corpus luteum

ADGRA3 (also known as GPR125; Fig. 1a) is an orphan member of the adhesion G protein-coupled receptor (ADGR/aGPCR) superfamily, belonging to the ADGRA subfamily (consisting of members ADGRA1–3) [36]. ADGRA3 was initially described as a marker for spermatogonial stem cells [37–39], and we recently identified it as a factor of male infertility due to ejaculatory duct obstruction in half of all male mice lacking ADGRA3 [40]. Moreover, Spina et al. recently described ADGRA3 expression on progenitor cells at the leading tips of the lacrimal ducts, which was required for proper tear film in mice, and on migrating progenitor cells at the invasive tips of ducts and branches during mammary development in mice [41, 42]. The direct cellular signaling mechanism for ADGRA3 has yet to be unraveled, but the receptor is expressed at the cell surface, where it constitutively internalizes [43]. ADGRA3 interacts directly through PDZ interactions with both Disheveled (DVL) and Discs large (DLG) [44–48], indicating potential roles in both canonical WNT and non-canonical planar cell polarity (PCP) pathways. Supporting this, previous studies have shown that ADGRA3 negatively regulates the canonical WNT/β-catenin pathway [49, 50], a pathway central to many developmental processes [51].

In this study, we aimed to investigate the role of ADGRA3 in female urogenital development and fertility. We hypothesized a similar role in females based on its role in the male reproductive system in mice [40] and its expression in spermatogonial stem cells in men [37–39]. We, therefore, disrupted endogenous *Adgra3* and found that 44% of ADGRA3-deficient females are infertile due to a lack of vaginal canalization at sexual maturity. Our data demonstrates a central role of ADGRA3 in distal vaginal tissue remodeling and formation of the vaginal opening.

## Results

### *Adgra3* is highly expressed in the prepubertal vagina and adult corpus luteum

To determine *Adgra3* expression in the normal female urogenital system, we performed qPCR on tissues from 8- to 10-week-old C57BL/6J female mice in proestrus. *Adgra3* was expressed ubiquitously in the urogenital system and endocrine tissues, including the pituitary and adrenal glands, with kidneys and ovaries expressing the highest levels and vaginae the lowest (Fig. 1b). In contrast, the other two ADGRA subclass members (*Adgra1* and *2*), though present in all tissues examined, were expressed principally in the pituitary gland and bladder (*Adgra1*) and to a lesser extent in the kidneys and ovaries (*Adgra2*; Additional file 1: Fig. S1a and b). *Adgra2* expression was similar to *Adgra3* in most other urogenital tissues (Fig. 1b and Additional file 1: Fig. S1b). Intriguingly, *Adgra3* was expressed to a higher extent in the ovaries (1.6-fold) and vagina (7-fold) of pre-pubertal mice (postnatal day 15, P15) compared to 8- to 10-week-old C57BL/6J mice (*p* = 0.0001 and *p* < 0.0001, respectively; Fig. 1c). Given the high ADGRA3 expression in spermatogonial stem cells [37–40], we investigated ADGRA3 within the ovaries utilizing transgenic mice expressing ADGRA3 fused to β-galactosidase at the first transmembrane domain [38, 40–42]. In these mice, ADGRA3 was predominantly located in the corpus luteum (Fig. 1d), with high expression in granulosa lutein cells (Fig. 1e and f).

### Imperforated vagina causes infertility in female mice lacking ADGRA3

**Fig. 2:**
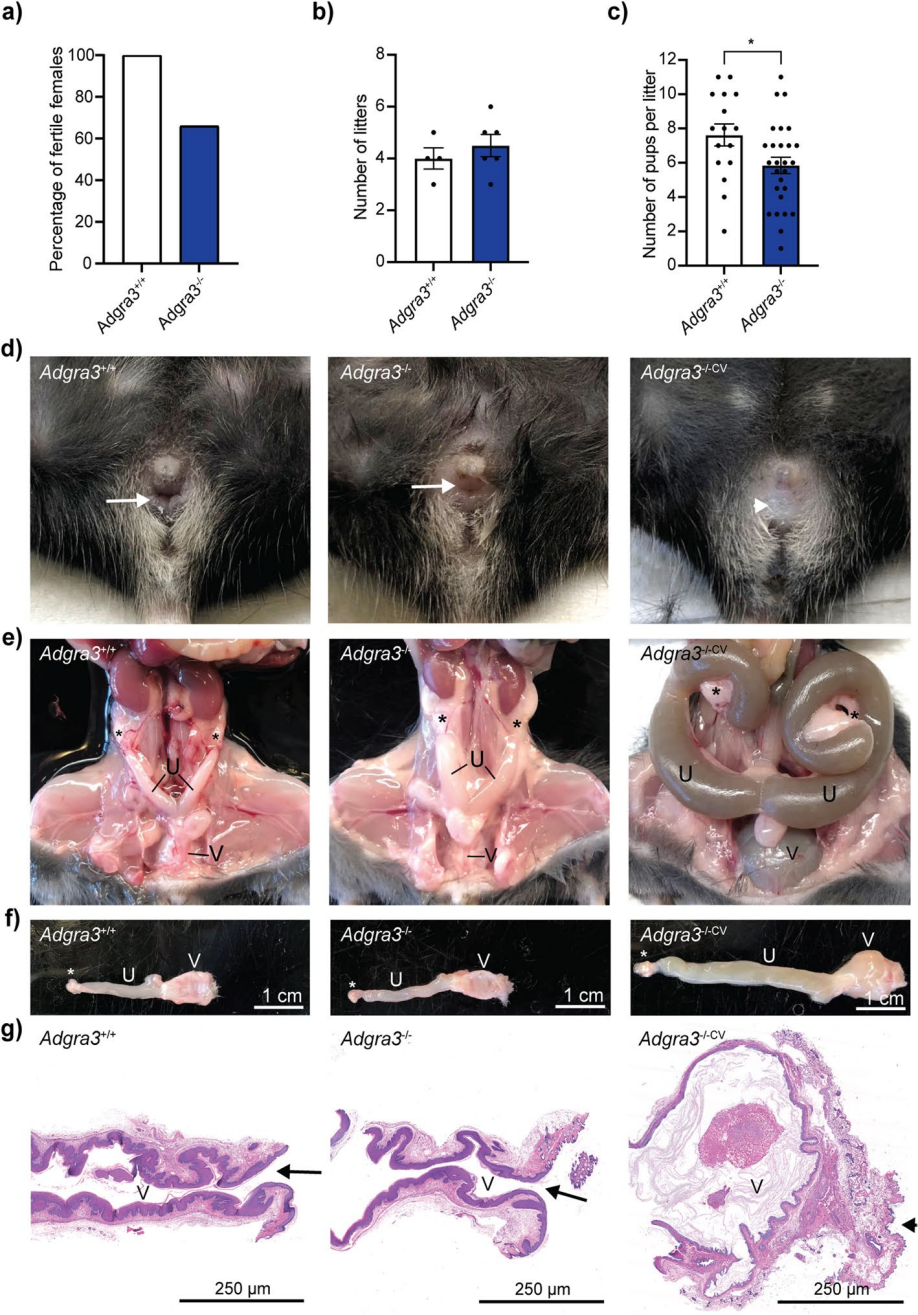
Infertility in *Adgra3*^*−/−*^ females is due to an imperforate vagina. **a** Percentage of fertile *Adgra3*^+*/*+^ (*n* = 4) and *Adgra3*^*−/−*^ (*n* = 9) females. **b** Number of litters per *Adgra3*^+*/*+^ and fertile *Adgra3*^*−/−*^ mothers after 5 months of consecutive breeding. Each dot represents an individual animal. **c** Number of pups in each litter from fertile animals (*p = 0.0312). Each dot represents one litter. Data are presented as mean ± SEM, and significance was determined by an unpaired *t*-test. **d**, **e** Representative images of vaginal openings (**d**) and female reproductive tracts (**e**) in 10-week-old *Adgra3*^+*/*+^, *Adgra3*^*−/−*^, and *Adgra3*^*−/−CV*^ mice (*n* = 3 per group). Arrow indicates an opened vaginal opening, and an arrowhead indicates a lack of vaginal opening observed in 44% of all *Adgra3*^*−/−*^ females in the colony. An asterisk (*) indicates the ovary. U, uterine horns; V, vagina. **f** Dissected female reproductive tract (from one ovary to the vagina) of 10-week-old *Adgra3*^+*/*+^ and *Adgra3*^*−/−*^ mice compared to the enlarged reproductive tract of a 10-week-old *Adgra3*^*−/−CV*^. **g** Hematoxylin and eosin (HE)-stained sections of the distal vagina in 10-week-old mice. Animals with an open vagina were euthanized after a smear test confirmed the proestrus stage

We generated an *Adgra3*^*−/−*^ mouse model on a C57BL/6J background by disrupting the endogenous *Adgra3* gene using the Cre-LoxP system and compared their fertility to that of littermate controls when paired with 8-week-old C57BL/6J males for 5 consecutive months. All control females were fertile, whereas only 6 out of 9 *Adgra3*^*−/−*^ females produced litters (Fig. 2a). Fertile *Adgra3*^*−/−*^ females produced a similar number of litters over 5 months as their *Adgra3*^+*/*+^ littermates (4.5 ± 0.43 vs. 4.0 ± 0.41 litters, *p* = 0.4468; Fig. 2b), but with significantly fewer pups per litter (6.0 ± 0.48 vs. 7.6 ± 0.65 pups/litter, *p* = 0.0312; Fig. 2c). Infertile *Adgra3*^*−/−*^ females were found to have a complete closure of the vaginal entrance, rendering the mice physically impenetrable (Fig. 2d). Overall, 44% (17/39) of *Adgra3*^*−/−*^ females had closed vaginae (hereafter referred to as *Adgra3*^*−/−CV*^). This phenotype was not observed in *Adgra3*^+*/*+^ (0/58) or *Adgra3*^+*/−*^ female littermates (0/100) (Additional file 2: Fig. 2a). Obstruction of the vaginal entrance resulted in intrauterine fluid accumulation, distention of the uterine horns (hydrometrocolpos) and vagina (hydrocolpos) (Fig. 2e), and gross enlargement of the female reproductive tract (Fig. 2f). Serial histological sectioning confirmed complete closure of the vaginal entrance in *Adgra3*^*−/−CV*^ females (Fig. 2g). The rest of the reproductive tract developed normally in all genotypes with no macroscopic or histological abnormalities. However, the pressure in the lumen distorted the vaginal and uterine histology of *Adgra3*^*−/−CV*^ females (Fig. 2g and Additional file 3: Fig. S3a-b). Moreover, the body weight of 8- to 10-week-old *Adgra3*^*−/−CV*^ females tended to be slightly higher, likely due to intrauterine fluid accumulation within the uterine horns, as the weight of other organs, such as the ovary, kidney, and brain, did not differ between genotypes (Additional file 3: Fig. S3c-g).

In addition to the imperforate vagina, all *Adgra3*^*−/−*^ mice of both sexes (86/86) exhibited a previously described ocular phenotype [41], which was not observed in either *Adgra3*^+*/−*^ (0/172) or *Adgra3*^+*/*+^ (0/97) mice (Additional file 2: Fig. S2).

### Ovarian morphology, estrus cycle, and puberty onset are unaltered despite ADGRA3-deficiency

**Fig. 3:**
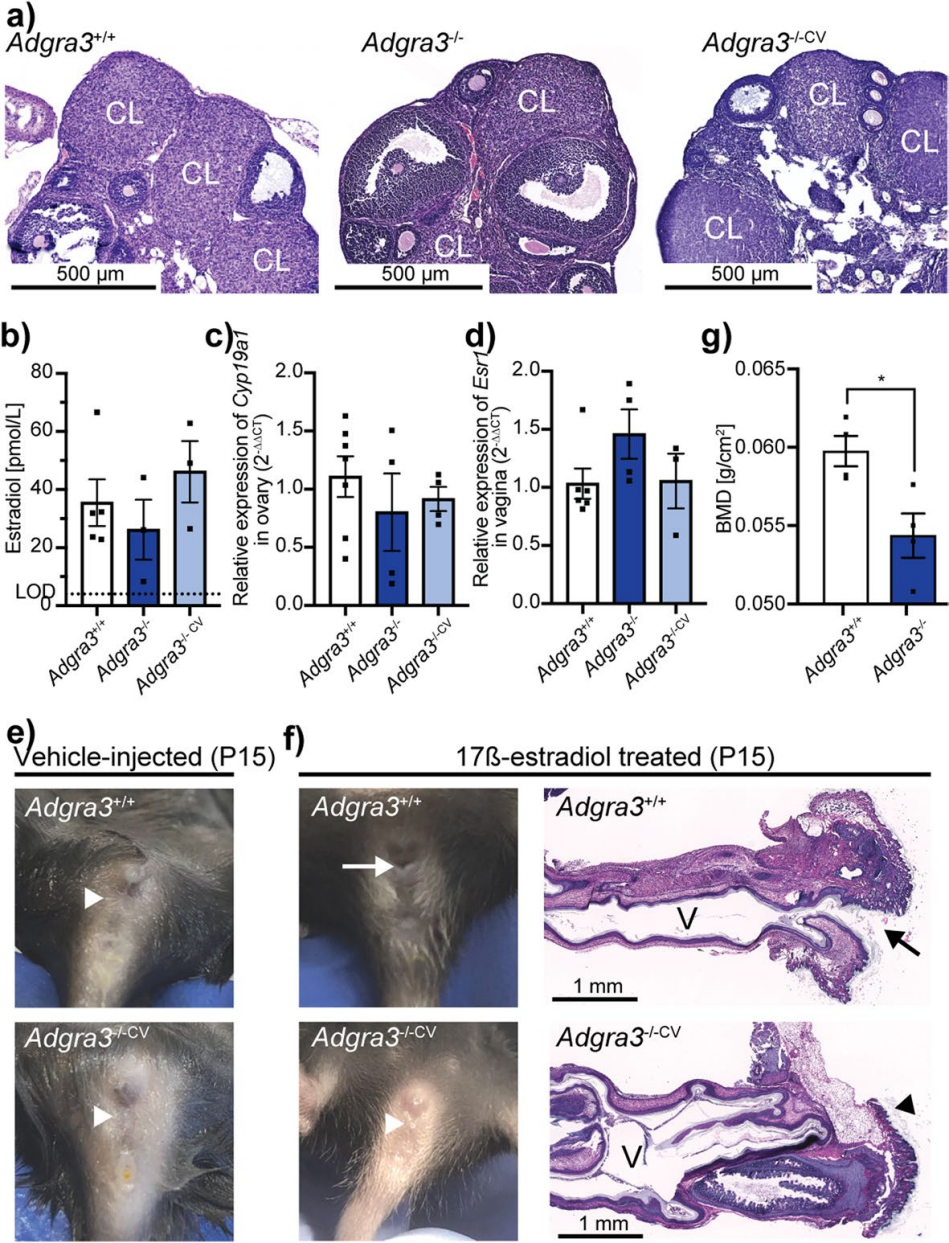
Ovarian morphology and function are unaltered despite the lack of ADGRA3, and estrogen treatment fails to induce vaginal opening in *Adgra3*^*−/−*^ mice. **a** Representative HE-stained sections of ovaries harvested from 10-week-old mice. Primary and secondary follicles and corpus luteum (CL) formation were observed in all *Adgra3*^+*/*+^ (*n* = 3), *Adgra3*^*−/−*^ (*n* = 3) in proestrus, and *Adgra3*^*−/−CV*^ (*n* = 3) mice analyzed. **b** Plasma estradiol levels in 8- to 10-week-old *Adgra3*^+*/*+^ (*n* = 5), *Adgra3*^*−/−*^ (*n* = 3), and *Adgra3*^*−/−CV*^ (*n* = 3) female mice. Limit of detection (LOD) = 4.04 pmol/L. **c** Relative mRNA expression of aromatase, *Cyp19a1*, in ovaries of 8- to 10-week-old *Adgra3*^+*/*+^ (*n* = 7), *Adgra3*^*−/−*^ (*n* = 4), and *Adgra3*^*−/−CV*^ (*n* = 4) mice. **d** Relative mRNA expression of estrogen receptor α (*Esr1*) in the vagina of 8- to10-week-old *Adgra3*^+*/*+^ (*n* = 6), *Adgra3*^*−/−*^ (*n* = 4), and *Adgra3*^*−/−CV*^ (*n* = 3) mice. **e** Pre-pubertal external genitalia of *Adgra3*^+*/*+^ and *Adgra3*^*−/−*^ mice at P15 (vehicle-injected). **f** Left: Genitalia of *Adgra3*^+*/*+^ and *Adgra3*^*−/−CV*^ at P15 after estrogen treatment. Right: Representative HE-stained sections of the distal vagina of *Adgra3*^+*/*+^ and *Adgra3*^*−/−CV*^ at P15 after estrogen treatment (*n* = 5 per group). Arrow indicates an opened vaginal opening, and an arrowhead indicates a lack of vaginal opening observed in 55% of *Adgra3*^*−/−*^ females after estrogen treatment. V, vagina. **g** Bone mineral density (BMD) measured in femurs harvested from *Adgra3*^+*/*+^ (*n* = 4) and *Adgra3*^*−/−*^ (*n* = 5) mice at 1 year of age. For ethical reasons, only bones from mice with an open vagina were investigated at year 1. Each dot represents one animal in all graphs. Data are presented as mean ± SEM, and significance was calculated by one-way ANOVA post hoc Dunnett’s multiple comparisons test for three groups and unpaired *t*-test of two groups. *p = 0.0198

In mice, vaginal opening is a hallmark of puberty, triggered by the first ovarian cycle and the corresponding increase in estrogen [11, 12]. As a measure of ovarian function, the onset of puberty was determined by the first day of the vaginal opening, which was similar between *Adgra3*^*−/−*^ (*n* = 5) mice and *Adgra3*^+*/*+^ (*n* = 14) littermates (28.2 ± 0.66 and 27.6 ± 0.67 days, *p* = 0.6480). Furthermore, the length of the estrus cycle and the frequency of each phase of the estrus cycle did not differ between *Adgra3*^*−/−*^ and *Adgra3*^+*/*+^ littermates (Additional file 3: Fig. S3h-i). These studies could not be performed on the infertile *Adgra3*^*−/−CV*^ females with closed vaginae. Nevertheless, by comparing the ovarian morphology of both *Adgra3*^*−/−*^ and *Adgra3*^*−/−CV*^ mice to that of *Adgra3*^+*/*+^ mice, we found no difference, as follicles in all stages, including the corpus luteum, were observed in ovaries from all three groups (Fig. 3a).

As estrogen is a main regulator of the vaginal opening, estradiol (E_2_) was analyzed in blood collected from 8- to 10-week-old females in the proestrus phase. No significant difference was observed between *Adgra3*^*−/−CV*^ and *Adgra3*^*−/−*^ vs. *Adgra3*^+*/*+^ mice (46.1 ± 10.6 and 26.2 ± 10.3 vs. 35.5 ± 8.04 pmol/L, *p* = 0.9695 and *p* = 0.6668 respectively; Fig. 3b). Similarly, the expression of ovarian aromatase, *Cyp19a1*, was comparable regardless of genotype (*Adgra3*^*−/−*^ and *Adgra3*^+*/*+^ with *p* = 0.9526 and *p* = 0.3089, respectively; Fig. 3c). Moreover, *estrogen receptor α* (*Esr1*) expression in vaginal tissue did not differ between *Adgra3*^*−/−CV*^ and *Adgra3*^+*/*+^ mice (*p* = 0.9991) or between *Adgra3*^*−/−*^ and *Adgra3*^+*/*+^ mice (*p* = 0.2192; Fig. 3d). Similarly, vaginal expression of genes encoding unconventional estrogen receptors (*Esr2* and *Gpr30*) did not differ between genotypes, regardless of vaginal phenotype (Additional file 4: Table S1).

### Estrogen treatment fails to induce vaginal opening in mice lacking ADGRA3

To exclude reduced estrogen as the cause of the imperforate vagina, the impact of exogenous estrogen treatment was investigated in pre-pubertal mice. Vehicle-injected *Adgra3*^+*/*+^ and *Adgra3*^*−/−*^ mice at P15 maintained a closed vagina (Fig. 3e), whereas all *Adgra3*^+*/*+^ had a fully formed vaginal entrance after 3 consecutive days of estrogen administration (Fig. 3f). However, the vagina remained closed in 55% (6/11) of *Adgra3*^*−/−*^ females (Fig. 3f), suggesting that the phenotype was not due to low endogenous estradiol production at puberty onset (55% vs. 44% in the original colony, *p* = 0.4811, *χ*^2^), but rather to a lack of estrogen responsiveness, even when circulating estrogen levels were high. In support of this, we observed significantly lower femoral bone mineral density (BMD), a long-term proxy of estrogen and estrogen signaling, in 1-year-old female *Adgra3*^*−/−*^ mice compared to *Adgra3*^+*/*+^ mice (0.054 ± 0.0014 vs. 0.060 ± 0.0010 g/cm2, *p* = 0.0198; Fig. 3g).

### Low progesterone receptor expression in vaginal tissue from adult mice with closed vagina

**Fig. 4:**
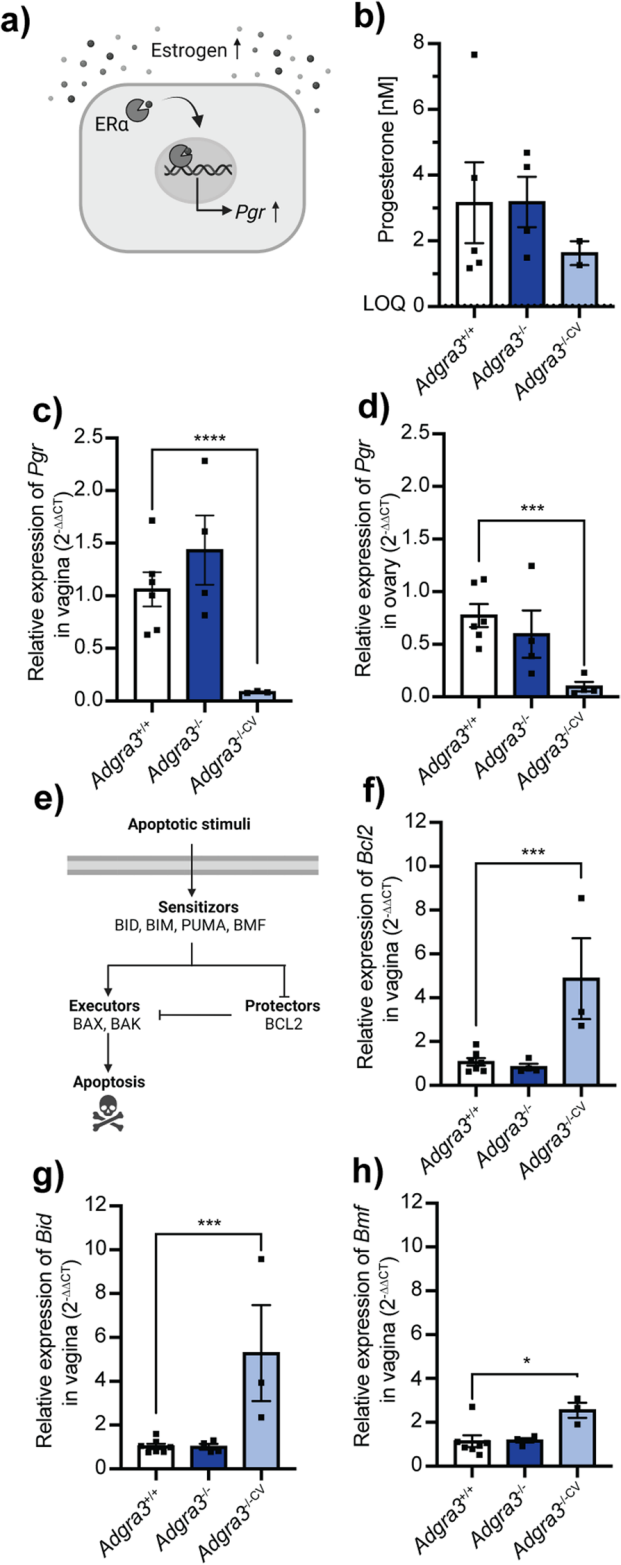
Lower progesterone receptor expression and increased expression of BCL2 family genes in *Adgra3*^*−/−*^ females with a closed vagina. **a** Illustration of the upregulation of progesterone receptor (*Pgr*) by estrogen receptor α (ERα) following estrogen stimulation in vaginal epithelial cells [52, 53]. **b** Progesterone levels measured in blood plasma harvested from 8- to 10-week-old *Adgra3*^+*/*+^ (*n* = 5), *Adgra3*^*−/−*^ (*n* = 4), and *Adgra3*^*−/−CV*^ (*n* = 3) mice in proestrus. Limit of detection (LOD) = 0.036 nmol/L. **c**, **d** mRNA expression of progesterone receptor (*Pgr*) in **c** vagina and **d** ovary of 8- to 10-week-old *Adgra3*^*−/−*^ (*n* = 4) and *Adgra3*^*−/−CV*^ (*n* = 3–4) mice relative to *Adgra3*^+*/*+^ (*n* = 6). ****p < 0.0001, ***p = 0.0002. **e** Illustration of the intrinsic apoptotic pathway regulated by the BCL2 family. Extracellular apoptotic stimuli activate sensitizer proteins, including BID, BIM, PUMA, and BMF. The sensitizers either inhibit the group of apoptotic protector proteins (e.g., BCL2) or activate apoptotic executors (e.g., BAX and BAK1). Activating apoptotic executor proteins leads to the release of cytochrome c from the mitochondria and subsequent apoptosis [54]. **f**–**h** mRNA expression of BCL2 family genes **f***Bcl2*, **g***Bid*, and **h***Bmf* in vaginal tissue from 8- to 10-week-old *Adgra3*^*−/−*^ (*n* = 4) and *Adgra3*^*−/−CV*^ (*n* = 3) mice relative to *Adgra3*^+*/*+^ (*n* = 7) mice. Relative expression was calculated using housekeeping genes *36b4* and *Ywhaz* and the 2^−∆∆CT^ method. ***p = 0.0009 (**f**), ***p = 0.0003 (**g**), *p* = 0.0129 (**h**). Each dot represents an individual animal. Data are presented as mean ± SEM. Significance was calculated using one-way ANOVA post hoc Dunnett’s multiple comparisons test

To determine if estrogen signaling was affected in the *Adgra3*^*−/−*^ mice, we investigated other well-known targets of estrogen signaling. Estrogen activation of estrogen receptor α (ERα) is an established regulator of progesterone receptor (*Pgr*) expression in the vaginal epithelium and stroma (Fig. 4a) [52, 53]. Therefore, we analyzed *Pgr* expression in vaginal tissue in combination with plasma progesterone. No significant change in progesterone was observed in 8- to 10-week-old *Adgra3*^*−/−CV*^ mice and *Adgra3*^*−/−*^ mice, compared with *Adgra3*^+*/*+^ littermates (1.6 ± 0.34 and 3.2 ± 0.77 vs. 3.2 ± 1.2 nM, *p* = 0.9990 and *p* = 0.7800, respectively; Fig. 4b). In contrast, vaginal *Pgr* expression was dramatically decreased (12-fold) in *Adgra3*^*−/−CV*^ mice compared with *Adgra3*^+*/*+^ mice (*p* < 0.0001) but not in *Adgra3*^*−/−*^ mice compared with *Adgra3*^+*/*+^ mice (*p* = 0.4347; Fig. 4c). Ovarian *Pgr* expression was 7-fold lower in *Adgra3*^*−/−CV*^ mice compared with *Adgra3*^+*/*+^ females (*p* = 0.0002), but this was not the case for *Adgra3*^*−/−*^ females (*p* = 0.5045; Fig. 4d). In addition, *Pgr* expression was significantly lower in pituitary glands and tended to be lower in uterine horns from *Adgra3*^*−/−CV*^ mice compared with *Adgra3*^+/^^+^ mice (Additional file 4: Table S1). No significant changes were observed in plasma progesterone (Additional file 5: Table S2) or vaginal *Pgr* expression between *Adgra3*^*−/−*^ and *Adgra3*^+*/*+^ mice before puberty (P15; Additional file 6: Table S3).

As the development of the female reproductive tract is a complex process with many contributing factors, we measured an array of steroid hormones and compared the expression patterns of relevant mRNA in pre-pubertal (P15) and mature (8- to 10-week-old) female mice (Additional files 4, 5 and 6: Tables S1-S3). No differences were found between genotypes regarding androgen levels (Additional file 5: Table S2) or expression of androgen receptor (*Ar*) or anti-Müllerian hormone receptor type 2 (*Amhr2*) at P15 or 8 to 10 weeks of age (Additional file 4: Table S1 and Additional file 6: Table S3). The anogenital distance (AGD), a measure of adequate androgen exposure, did not differ between *Adgra3*^*−/−*^, *Adgra3*^*−/−CV*^, and *Adgra3*^+*/*+^ mice at any age (Additional file 7: Fig. S4).

### BCL2 family genes are upregulated in vaginal tissue from mice with imperforate vagina

Progesterone promotes epithelial apoptosis in the vagina [53]. As the intrinsic apoptosis pathway is controlled by the B-cell lymphoma 2 (BCL2) family of proteins (Fig. 4e) [54, 55], we investigated the expression of selected BCL2 family genes. We found a marked upregulation of *Bcl2* in the vaginal tissue of mice with closed vagina (approximately 5-fold higher in 8- to 10-week-old *Adgra3*^*−/−CV*^) compared to *Adgra3*^+*/*+^ mice (*p* = 0.0009; Fig. 4f). Concurrently, higher expression of genes encoding apoptotic sensitizers, *Bid* (5-fold) and *Bmf* (3-fold), was found in vaginal tissue from *Adgra3*^*−/−CV*^ mice compared with *Adgra3*^+*/*+^ mice (*p* = 0.0003 and *p* = 0.0129, respectively; Fig. 4g, h). Importantly, the expression of all BCL2 family genes measured in vaginal tissue was not different between *Adgra3*^*−/−*^ and *Adgra3*^+*/*+^ mice (Fig. 4f–h and Additional file 4: Table S1). Furthermore, before pubertal onset (P15), *Bcl2*, *Bid*, *Bmf*, *Bax*, and *Bak1* were all expressed similarly in the vaginal tissue of *Adgra3*^*−*/*−*^ and *Adgra3*^+/+^ mice (Additional file 6: Table S3). Other timed postnatal remodeling processes, such as eye-opening and release of the ear flap, were normal in all mice. No interdigital webs were observed in any mice during this study. Thus, changes in apoptosis factors correlated temporally with the manifestation of the closed vagina phenotype.

## Discussion

Congenital abnormalities of the female reproductive tract are common and have severe consequences, but their etiologies are often unknown [7]. Here, we report that ADGRA3 significantly influences female fertility and is crucial for normal vaginal canalization. Approximately half of the ADGRA3-deficient females failed to develop a vaginal opening during puberty, a phenotype found rarely (0.7%, 1/154) in wild-type C57BL/6 females [56]. *Adgra3* is expressed throughout the female urogenital system, with higher expression in pre-pubertal mice, which drops precipitously after sexual maturation. This is consistent with patterns of *Adgra3* expression in the lacrimal and mammary glands, which also undergo significant postnatal development, where expression is associated with undifferentiated cell states and migratory processes associated with concurrent lumen formation [41, 42].

In mice, vaginal opening correlates with a pubertal increase in plasma estradiol [12]. However, we found no significant difference between adult *Adgra3*^*−/−*^ mice and littermate controls regarding puberty onset, estrus cycling, plasma estradiol, ovarian morphology, or *Cyp19a1* expression. Moreover, exogenous estradiol treatment failed to rescue the vaginal phenotype in *Adgra3*^−*/*−*CV*^ females. Collectively, these results exclude reduced estrogen levels at puberty as the cause of the imperforate vagina. Furthermore, vaginal expression of estrogen receptors (*Esr1*, *Esr2,* and *Gpr30*) remained unchanged, indicating that the vaginal tissue’s ability to receive estrogen signals remained intact in *Adgra3*^*−/−CV*^ mice. Nevertheless, ADGRA3 deficiency was associated with a significant reduction in established hallmarks of long-term estrogen signaling, such as femoral BMD, consistent with previous studies [40]. However, as ADGRA3 was recently described as a positive regulator of osteoclastogenesis [57], and normal osteoclast activity is needed for dynamic bone remodeling, it is possible that the impaired BMD was also affected directly by the low ADGRA3 expression.

Estrogen has been linked to progesterone signaling in the uterus, ovaries, and mammary glands [58, 59] and is a known inducer of *Pgr* expression in the vaginal epithelium and stroma [52, 53]. The marked reduction in vaginal *Pgr* mRNA expression in adult *Adgra3*-knockout mice with closed vaginae (*Adgra3*^*−/−CV*^) but not in those with open vaginae or in wild-type littermate controls supports an overall impairment of the downstream effectors of the estrogen axis. Notably, the highest expression of *Adgra3* was found in the ovary, specifically in the corpus luteum, the location of progesterone production [60]. As the primary role of progesterone is to support the embryo and secure implantation in the uterus [60], a suboptimal progesterone axis in *Adgra3*^*−/−*^ dams during gestation could explain their smaller litter size. In support of a connection between ADGRA3 and progesterone signaling, the ocular phenotype observed *in Adgra3*^*cre/cre*^ and *Adgra3*^*lz/lz*^ mice exacerbates in dams during pregnancy when progesterone levels are high but resolves during weaning [41]. However, downregulation of sex hormone receptors is not sufficient to drive an impairment of vaginal canalization, as evident from no reports of imperforate vagina in single- and double-knockout of *Esr1* and *Esr2* [61] or *Pgr*-null mice, though these mice have severe fertility issues [58]. This suggests that other factors, driven by the lack of ADGRA3, contribute to the observed vaginal phenotype in our mice.

Although the exact mechanism of estrogen-driven apoptosis during vaginal canalization is unknown, progesterone signaling has been linked to apoptosis in the vaginal epithelium [53]. We observed no significant difference in apoptosis factors during the prepubertal period but found an overall increase in vaginal *Bcl2*, *Bid*, and *Bmf* expression in 8- to 10-week-old *Adgra3*^*−/−CV*^ mice. Due to the accumulated fluid within the reproductive tract, the upregulation of *Bcl2*, *Bid*, and *Bmf* may have been secondary to the pressure within the lumen rather than a direct loss of *Adgra3*. However, a 1.4-fold increase in vaginal *Bcl2* expression was observed in *Adgra3*^*−/−*^ at P15 (Additional file 6: Table S3). Although non-significant, this timing supports the concept that ADGRA3 may function to regulate the balance between pro- and anti-apoptotic signals during vaginal canalization. Several apoptotic genes are associated with vaginal aberrations in mice. For example, triple-knockout of apoptotic sensitizers *Bid*, *Bim*, and *Puma* or dual knockout of pro-apoptotic genes *Bak* and *Bax* causes imperforate vagina in 100% of knockout mice, and this phenotype also occurs with lower penetrance in 20% of *Bmf-*knockout mice [24–26]. Furthermore, 100% of transgenic mice overexpressing the anti-apoptotic gene *Bcl2* have a closed vagina in adulthood and, similar to our findings in *Adgra3*^*−/−CV*^ mice, cannot be rescued by estrogen treatment [11]. Thus, in contrast to targeted deletion of the sex hormone receptors, which is insufficient to drive imperforate vagina development, changes in apoptotic regulators are strong enough to drive this phenotype.

Development of the Müllerian ducts and female reproductive tract is highly dependent on proper WNT signaling [9, 15], as evident from imperforate vagina having a reported penetrance of 30% to 100% in knockout experiments of other genes involved in WNT signaling (e.g., *Ctnnb1*, *Wnt4* and *Wnt5a*, *Dlg1*, *Vangl2*, *Map3k1*, *Ovo1*) [16–21]. Notably, a missense mutation in β-catenin results in a similar phenotype in male mice, as we previously described for *Adgra3*^*−/−*^ males [40], in addition to the imperforate vagina in female mice [16]. At the signaling level, it is still unknown whether ADGRA3 signals via G proteins. However, several groups have reported a strong linkage between ADGRA3 and WNT signaling, focusing on both the developmental and other roles of this receptor [44–50]. A similar linkage with WNT has also been reported for the closely related ADGRA2 [62]. Similar to ADGRA3, ADGRA2 is required for tip cell function, although in endothelial cells during angiogenesis in the brain [62]. Intriguingly, vaginal *Adgra2* expression is slightly higher in P15 *Adgra3*^*−/−*^ mice and significantly higher in 8- to 10-week-old *Adgra3*^*−/−CV*^ mice compared to littermate controls (Additional file 4: Table S1 and Additional file 6: Table S3). Further investigation is required to determine if ADGRA2 compensates for the loss of ADGRA3 and if this is the reason for the incomplete penetrance of the vaginal phenotype.

## Conclusions

Our findings suggest that ADGRA3-deficiency impairs the estradiol-dependent vaginal canalization via an altered balance in apoptotic regulators, potentially driven by WNT signaling. Future efforts will be directed at deciphering if and how ADGRA3 and ADGRA2 act in concert to affect WNT-driven epithelial remodeling in the distal vagina. As G protein-coupled receptors are excellent drug targets, our study provides support for investigating ADGRA3 as a target in diagnostic screening and/or therapy for obstructive vaginal malformations in humans.

## Methods

### Animals and housing

*Adgra3*^*lz/*+^ mice were described by Seandel et al. [38] and Spina et al. [41, 42]. *Adgra3*^*−/−*^ and *Adgra3*^+*/*+^ mice were generated from heterozygous breeding and were used previously in Nybo et al. [40]. All mice were housed in individually ventilated cages with 8–10 air changes per hour. The environment was maintained at 22 °C (± 2 °C) and 55% (± 10%) air humidity. The stables maintained a 12:12-h light-to-dark cycle. The mice received an ad libitum chow diet (D30, SAFE, Rosenberg, Germany) and had free access to tap water. Housing, breeding, and experiments were conducted according to institutional guidelines and approved by the Animal Experiments Inspectorate under the Danish Ministry of Food, Agriculture, and Fisheries. License numbers for this study were 2017-15-0202-00117, 2017-15-0201-01235, and 2018-15-0201-01442.

### Genotyping

Genotypes were determined using genomic DNA from ear or tail biopsies. Tissue samples were lysed in boiling (90–95 °C) 25 mM sodium hydroxide (NaOH) and 0.2 mM Ethylenediaminetetraacetic acid (EDTA) solution for 30 min, and DNA stabilized in 40 mM Tris-Hydrochloride. Three primers (forward P2: CTGAAGTTCTTTTGACAGAATCTCGGCAC, forward P5: AGAACCATGAAACGTGGGATACCTGTTTC, reverse P3: TGGAGCATACACGAGCACTCTGTTA GTCA) were used to identify the wild-type (200 bp) and mutated alleles (500 bp). PCR was performed using PCR Master Mix (2X) (Thermo Fisher Scientific, Vilnius, Lithuania) according to the manufacturer’s protocol. Primer products were amplified using an Eppendorf Mastercycler EP Gradient S Thermal Cycler with initial denaturing at 94 °C for 2 min, followed by 30 cycles of 94 °C for 30 s, 65 °C for 30 s, and 68 °C for 2 min, and completed at 68 °C for 8 min. PCR products were loaded into 1% agarose gel containing 1× GelRed Nucleic Acid stain (10000X) (Biotium, Fremont, CA, USA) alongside 1 kb Plus DNA Ladder (Thermo Fisher Scientific, Vilnius, Lithuania) and separated by electrophoresis. Products were detected in the gel using 1× Gel Loading Dye Purple (6X) (New England BioLabs, MA, USA), and PCR products were visualized using a Protein Simple SA-1000 (Red) gel imager.

### Fertility trial

The fertility of female mice was studied by housing 8-week-old *Adgra3*^*−/−*^ and *Adgra3*^+*/*+^ mice together with 8-week-old wild-type males for 5 months. Breeding pairs of four (one female *Adgra3*^+*/*+^, two female *Adgra3*^*−/−*^*,* and one male *Adgra3*^+*/*+^) were caged together. *Adgra3*^+*/*+^ females were housed with males 5 days ahead of *Adgra3*^*−/−*^ females to prevent simultaneous birthing. Litter sizes were monitored at birth during the study period.

### Monitoring puberty onset and estrus cycle

To determine the onset of puberty, the status of the vaginal opening was evaluated each morning from P20 until complete formation or P35. The estrus cycle stage was ascertained for 17 consecutive days from the age of 7 weeks by vaginal lavage and vaginal cytology as described previously [63]. Briefly, the vagina was flushed with 100 μl saline, and the smear was air-dried, stained for 1 min in 0.02% crystal violet in absolute ethanol, and rinsed twice for 1 min in distilled water. The stages of the estrus cycle were defined by the presence of primarily anucleate cornified cells (estrus), a combination of anucleate cornified cells and leukocytes (metestrus), mostly leukocytes (diestrus), or the presence of primarily small, nucleated cells (proestrus). Vaginal lavage was performed in the morning between 9 and 11 am and in the evening between 8 and 10 pm, as the metestrus and proestrus stages of the cycle last less than 24 h in mice [63].

### Pre-pubertal estradiol treatment

In mice, canalization of the vagina occurs at puberty via an estrogen-mediated induction of apoptosis of the distal vaginal epithelium to generate the vaginal entrance [10, 11]. Thus, the opening of the murine vagina remains closed at birth and is sensitive to local and systemic alterations in estrogen signaling that accompany sexual maturation [12–14]. Pre-pubertal mice were injected intraperitoneally with 2.5 μg/g 17β-estradiol (Sigma-Aldrich. St. Louis, MO, USA) dissolved in isopropanol and diluted in flax seed oil for three consecutive mornings starting from postnatal day 12 (P12). The status of the vaginal opening was assessed and recorded each morning.

### Anogenital distance (AGD)

The AGD of female *Adgra3*^*−/−*^ mice and control littermates was assessed by photograph, allowing for repeated assessment. The mice were placed on a Plexiglas platform with a built-in measuring tape and a digital camera approximately 10 cm below. The mice were held stationary by the tail and photographed. These photographs were depicted at high magnification and analyzed by two blinded investigators. The AGD was measured as the length of the perineum from the center of the genital papilla to the proximal end of the rectum [64].

### Blood sampling, tissue preparation, and qPCR

Adult animals were euthanized by cervical dislocation during the proestrus stage. Blood was drawn from the main aorta immediately after decapitation, and organs were harvested and snap-frozen in liquid nitrogen or immersed in 3.7% formaldehyde in phosphate-buffered saline (PBS). Plasma was separated from whole blood by centrifugation at 1000 rpm at 4 °C for 15 min. The right femur was cut free from the muscles, fixated in 70% ice-cold ethanol, and stored at 4 °C until further analysis. Total RNA was extracted from snap-frozen tissue using the RNeasy Mini Kit (Qiagen, Hilden, Germany) or RNeasy Micro Kit (Qiagen, Hilden, Germany) for tissues > 5 mg or < 5 mg, respectively, according to the manufacturer’s protocol. RNA was measured using NanoDrop One (Thermo Fisher Scientific, Wilmington, DE, USA), and reverse transcription of purified RNA (2000 ng) was performed using the High-Capacity cDNA Reverse Transcription Kit (Applied Biosystems, Foster City, CA, USA) according to the manufacturer’s instructions. Real-time quantitative PCR (qPCR) analyses were performed using the Quantstudio 6 Flex Real-Time PCR System (Applied Biosystems, Forster City, CA, USA) and PrecisionPLUS qPCR Master Mix with low ROX and SYBRGreen (Primer Design, Chandler’s Ford, UK). All primers were used at a concentration of 250 nM per 10 μl reaction, and cDNA was used at 1 ng/μl. Gene expression was reported relative to housekeeping genes tyrosine 3/tryptophan 5-monooxygenase activation protein, zeta polypeptide (*Ywhaz*), and ribosomal protein, large, P0 (*36b4/Rplp0*), and was calculated by the Livak (2^−ΔΔCt^) method (for two or more groups, normalized to *Adgra3*^+*/*+^) or the 2^−ΔCt^ method (for one group). Primers were ordered from TAG Copenhagen (Frederiksberg, Copenhagen), and their efficiencies were established according to MIQE guidelines [65]. Primers with efficiency (E%) between 90 and 110% were used. A complete list of primer details is provided in Additional file 8: Table S4.

### Dual-energy X-ray absorptiometry (DEXA)

Femora from 1-year-old *Adgra3*^+*/*+^ and *Adgra3*^*−/−*^ females underwent densitometry using a DEXA scanner (Sabre XL, Norland Stratec, Pforzheim, Germany). The entire femur was scanned at a pixel size of 0.1 × 0.1 mm and a velocity of 3.0 m/s. The BMD was determined using the software supplied with the scanner. Quality control was conducted using two solid-state phantoms.

### Liquid chromatography with tandem mass spectrometry (LC–MS/MS)

Serum concentrations of estrone, estradiol, estrone 1-sulfate, cortisone, cortisol, dehydroepiandrosterone sulfate (DHEAS), corticosterone, 11-deoxycortisol, ∆4-androstenedione, testosterone, 17ɑ-hydroxyprogesterone, and progesterone were analyzed by sensitive isotope dilution TurboFlow-LC-MS/MS methods for simultaneous quantification of estrogens [66] and androgens/corticosteroids [67]. Although these methods were developed for human serum, they have previously been used without modification in mice [68]. Samples were evaluated across six batches, each including standards for calibration curves, one blank, three un-spiked serum pool samples, three pool controls spiked with a high concentration, and three pool controls spiked with a low concentration. The matrix for control material was pooled human serum from prepubertal children and adults. In the six batches, the inter-day variation (CV%) in control material ranged from 1.4 to 8.2% for estrogens and 0.8 to 5.4% for the androgens/corticosteroids and were within the common inter-day variation for batches including human patient samples. The limit of detection (LOD) and limit of quantification (LOQ) for the steroids were 2.93 pmol/L (estrone), 4.04 pmol/L (estradiol), 0.026 nmol/L (estrone 1-sulfate), 0.19 nmol/L (cortisone), 1.9 nmol/L (cortisol), 19 nmol/L (DHEAS), 0.1 nmol/L (corticosterone), 0.017 nmol/L (11-deoxycortisol), 0.1 nmol/L (17ɑ-hydroxyprogesterone), 0.012 nmol/L (testosterone), 0.042 nmol/L (∆4-androstenedione), and 0.036 nmol/L (progesterone) when the same control material was used.

### Hematoxylin and eosin staining

Formalin-fixed tissues were paraffin-embedded, sectioned (4- to 5-μm), mounted on glass slides, and stained at the Histolab, Core Facility of Integrated Microscopy (Biomedical Institute, University of Copenhagen, Denmark). Hematoxylin and eosin staining was used to determine organ and tissue morphology. Serial sections were used to determine the nature and extent of vaginal obstruction. Images were acquired using an Axio Scan.Z1 (brightfield) Slide Scanner (Carl Zeiss, Jena, Germany) and Zeiss Zen Blue 3.0 Software (Carl Zeiss, Jena, Germany).

### X-gal staining

Ovaries from adult female *Adgra3*^*lz/*+^ and control mice were fixed in 4% paraformaldehyde (PFA) for 30 min at room temperature, washed in PBS, and rinsed for 1 h in X-gal rinse buffer (0.2% NP-40, 2 mM MgCl_2_, 0.1% sodium deoxycholate in PBS). Tissues were incubated at 4 °C for 14 h in X-gal staining solution (50 mg/ml 5-bromo-4-chloro-3-indolyl-β-D-galactopyranoside; PanReac AppliChem, Darmstadt, Germany) in rinse buffer containing 5 mM potassium ferricyanide, followed by a wash with PBS and overnight post-fixation in 4% PFA. Tissues were paraffin-embedded and sectioned into 10-μm slices for histological evaluation.

### Statistical analysis

All statistical analyses were performed in Prism 9 (Graph Pad Inc., San Diego, CA, USA). The qPCR data (values from cycle threshold) were normalized to internal controls *Ywhaz* and *36b4*. Outliers in qPCR analyses were identified using the ROUT method with a false discovery rate of *Q* = 1%. Outliers were excluded from further analyses and figures. Calculations were based on ΔCT values for qPCR data. Before statistical analysis, the normality of data distribution and homogeneity of variances were assessed using Shapiro-Wilk tests and Levene or Bartlett tests, respectively. Variance and *p*-values were calculated using a two-tailed, unpaired Student *t*-test (two groups) or one-way ANOVA followed by Dunnett’s multiple comparisons test for post hoc analysis (three groups). When the sample size was too small to assess normal distribution, the non-parametric test of Kruskal-Wallis, followed by Dunn’s multiple comparisons test, was performed to provide a mean of statistical analysis without assuming a specific distribution. Observed vs. expected characteristics (observed closed vagina with vs. without estradiol treatment) were compared using the *χ*^2^ test. Continuous outcomes are presented as mean ± SEM, and significance was defined as *p* ≤ 0.05.

### Supplementary Information

**Additional file 1: Figure S1. ***Adgra*1 and *Adgra2* expression in the female mouse urogenital tract. (a-b) Expression pattern of (a) *Adgra1* and (b) *Adgra2* in the urogenital system of 8- to 10-week-old C57BL6/J female mice in proestrus (*n* = 4). Relative expression was calculated relative to housekeeping genes *36b4* and *Ywhaz* using the 2^-∆CT^ method. Data are presented as mean±SEM.

**Additional file 2: Figure S2.** Observed phenotypes in *Adgra3*^*-/-*^ mutant mice. (a) Incidence of phenotypic traits: imperforate vagina, eye phenotype, and kinked tail. (b) Representation of the eye phenotype observed in all *Adgra3*^*-/-*^ mice. (c) A representative image of the kinked tail observed in 14% of *Adgra3*^*-/-*^ mice. The kinky tail occurrence did not co-segregate with the imperforate vagina and was not further investigated.

**Additional file 3: Figure S3.** Ovarian and uterine morphology, weight of adult organs, body weight, and estrus cycle of experimental groups. (a) HE-stained whole ovary sections from *Adgra3*^*+/+*^, *Adgra3*^*-/-*^, and *Adgra3*^*-/-CV*^ mice. (b) HE-stained sections of the middle part of the right uterine horn in *Adgra3*^*+/+*^, *Adgra3*^*-/-*^, and *Adgra3*^*-/-CV*^ mice. The uterine morphology is disturbed in *Adgra3*^*-/-CV*^, possibly due to the large fluid accumulation within the uterine horns. (c-f) Weight of (c) ovary, (d) uterine horn, (e) kidney, and (f) brain collected from 8- to 10-week-old *Adgra3*^*+/+*^ (*n* = 9), *Adgra3*^*-/-*^ (*n* = 5), and *Adgra3*^*-/-CV*^ (*n* = 3) mice. (g) Body weight of the age subgroups used in this study. P15: *Adgra3*^*+/+*^ (*n* = 5) and *Adgra3*^*-/-*^ (*n* = 6); 8- to 10 weeks: *Adgra3*^*+/+*^ (*n* = 9), *Adgra3*^*-/-*^ (*n* = 5), *Adgra3*^*-/-CV*^ (*n* = 3); 1 year: *Adgra3*^*+/+*^ (*n* = 9) and *Adgra3*^*-/-*^ (*n* = 9). Each dot represents an individual animal. (h) The average length of each estrus cycle of *Adgra3*^*+/+*^ (*n* = 6) and *Adgra3*^*-/-*^ (*n* = 4) mice with an open vagina. Each dot represents one cycle counted as the days from one proestrus to the next. (i) The frequency of estrus, diestrus, proestrus, and metestrus observed in *Adgra3*^*+/+*^ (*n* = 6) and *Adgra3*^*-/-*^ (*n* = 4) females with an open vagina are presented as the number of times each phase was recorded over 17 days. Data are presented as mean ±SEM. ****p < 0.0001.

**Additional file 4: Table S1.** Endogenous expression of select genes in the ovary, vagina, uterine horn, and pituitary gland whole tissue from *Adgra3*^*-/-*^ and *Adgra3*^*-/-CV*^ mice compared to *Adgra3*^*+/+*^ mice at 8-10 weeks.

**Additional file 5: Table S2.** Plasma hormone levels collected from *Adgra3*^*+/+*^, *Adgra3*^*-/-*^, and *Adgra3*^*-/-CV*^ females at 8–10 weeks and P15.

**Additional file 6: Table S3.** Endogenous expression of select genes in the ovary, vagina, uterus, and pituitary of *Adgra3*^*-/-*^ mice compared to *Adgra3*^*+/+*^ mice at P15.

**Additional file 7: Figure S4.** Anogenital distance in ADGRA3-deficient females compared to wild-type at postnatal day 4, 4–5 weeks, 8–10 weeks, and 1 year of age. Anogenital distance measured between the rectum and the center of the genital papilla in 4-day-old (*Adgra3*^*+/+*^*n* = 7, *Adgra3*^*-/-*^*n* = 7), 4 to 5-week-old (*Adgra3*^*+/+*^*n* = 10, *Adgra3*^*-/-*^*n* = 4), 8- to 10-week-old (*Adgra3*^*+/+*^*n* = 12, *Adgra3*^*-/-*^*n* = 6, *Adgra3*^*-/-CV*^*n* = 2), and 1-year-old females (*Adgra3*^*+/+*^*n* = 7, *Adgra3*^*-/-*^*n* = 5, *Adgra3*^*-/-CV*^*n* = 2). Each dot represents an individual animal.

**Additional file 8: Table S4.** Primer details.

## Data Availability

All data generated and analyzed during this study are included in this published article and its supplementary information. Information and requests for resources and reagents should be directed to the lead contact, Mette M. Rosenkilde (rosenkilde@sund.ku.dk). Due to institutional policy, the mouse lines in this study will be shared upon request after a Material Transfer Agreement is completed.

## Abbreviations

7TM: Seven transmembrane
ADGR/aGPCR: Adhesion G-protein coupled receptor
ADGRA3: Adhesion G-protein coupled receptor A3
AGD: Anogenital distance
AMH: Anti-Müllerian hormone
AMHR: Anti-Müllerian hormone receptor
AR: Androgen receptor
BCL2: B-cell lymphoma 2
BMD: Bone mineral density
DHEAS: Dehydroepiandrosterone sulfate
DLG: Discs large
DVL: Dishevelled
E_2_: Estradiol
ERα: Estrogen receptor alpha
GAIN: GPCR auto-proteolysis inducing
HBD: Hormone binding domain
Ig-like: Immunoglobulin-like
LC-MS/MS: Liquid chromatography with tandem mass spectrometry
LoxP: Locus of X-over, P1
LRR: Leucine-rich repeats
P: Postnatal day
PBM: PDZ-binding domain
PBS: Phosphate-buffered saline
PCP: Planar cell polarity
PDZ: Post-synaptic density protein-95, Drosophila discs large and zonula occludens-1
PGR: Progesterone receptor
WNT: Wingless-related MMTV integration site
