# Supplementary figures and images for: High incidence of imperforate vagina in ADGRA3-deficient mice
Source: BMC Biol. 2024 Apr 8;22:77. doi: 10.1186/s12915-024-01873-6 (PMC11003089; doi:10.1186/s12915-024-01873-6)

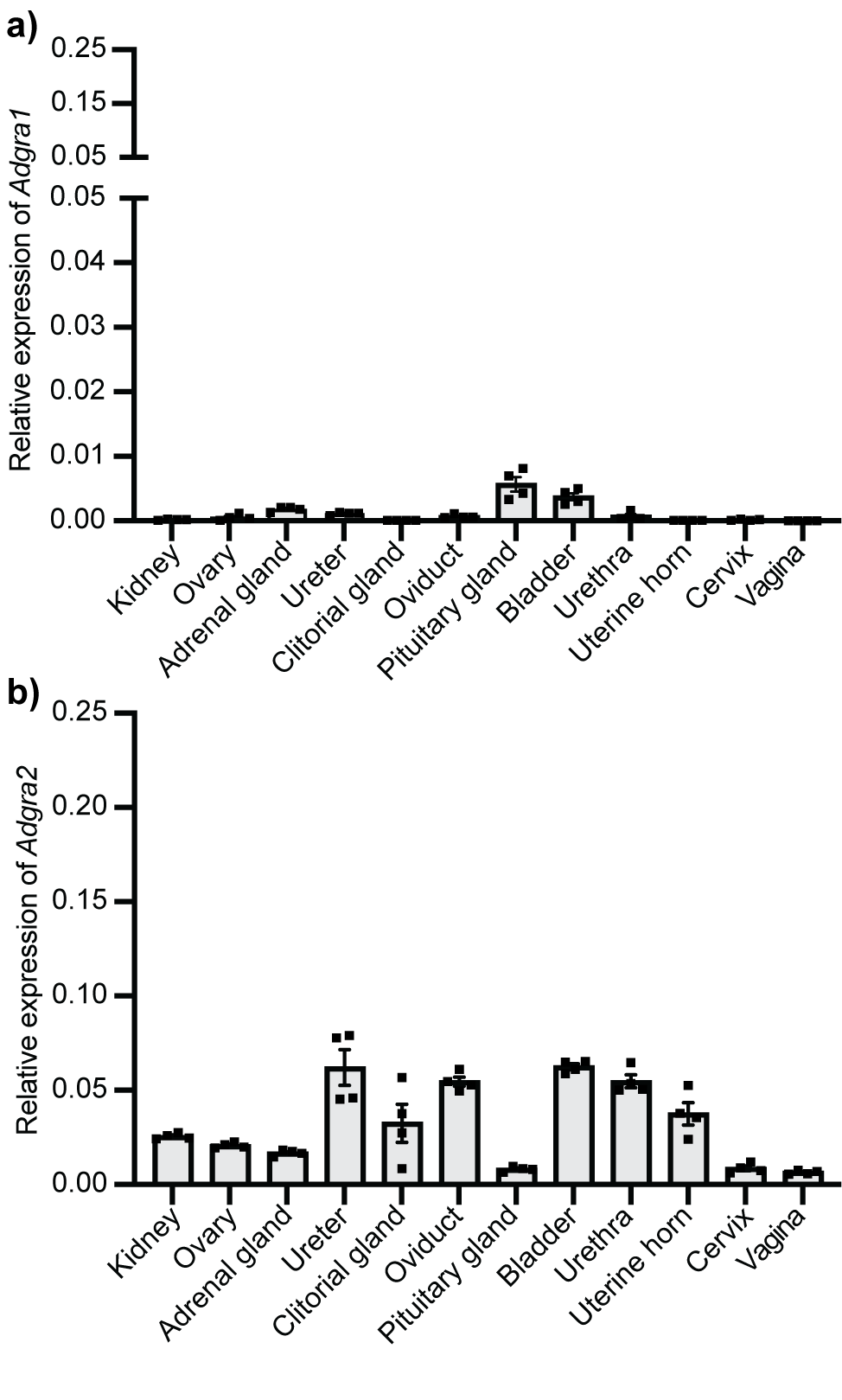

Supplement: Supplementary file 1 — Additional file 1: Figure S1. Adgra1 and Adgra2 expression in the female mouse urogenital tract. (a-b) Expression pattern of (a) Adgra1 and (b) Adgra2 in the urogenital system of 8- to 10-week-old C57BL6/J female mice in proestrus (n = 4). Relative expression was calculated relative to housekeeping genes 36b4 and Ywhaz using the 2-∆CT method. Data are presented as mean±SEM. [file 12915_2024_1873_MOESM1_ESM.tif]

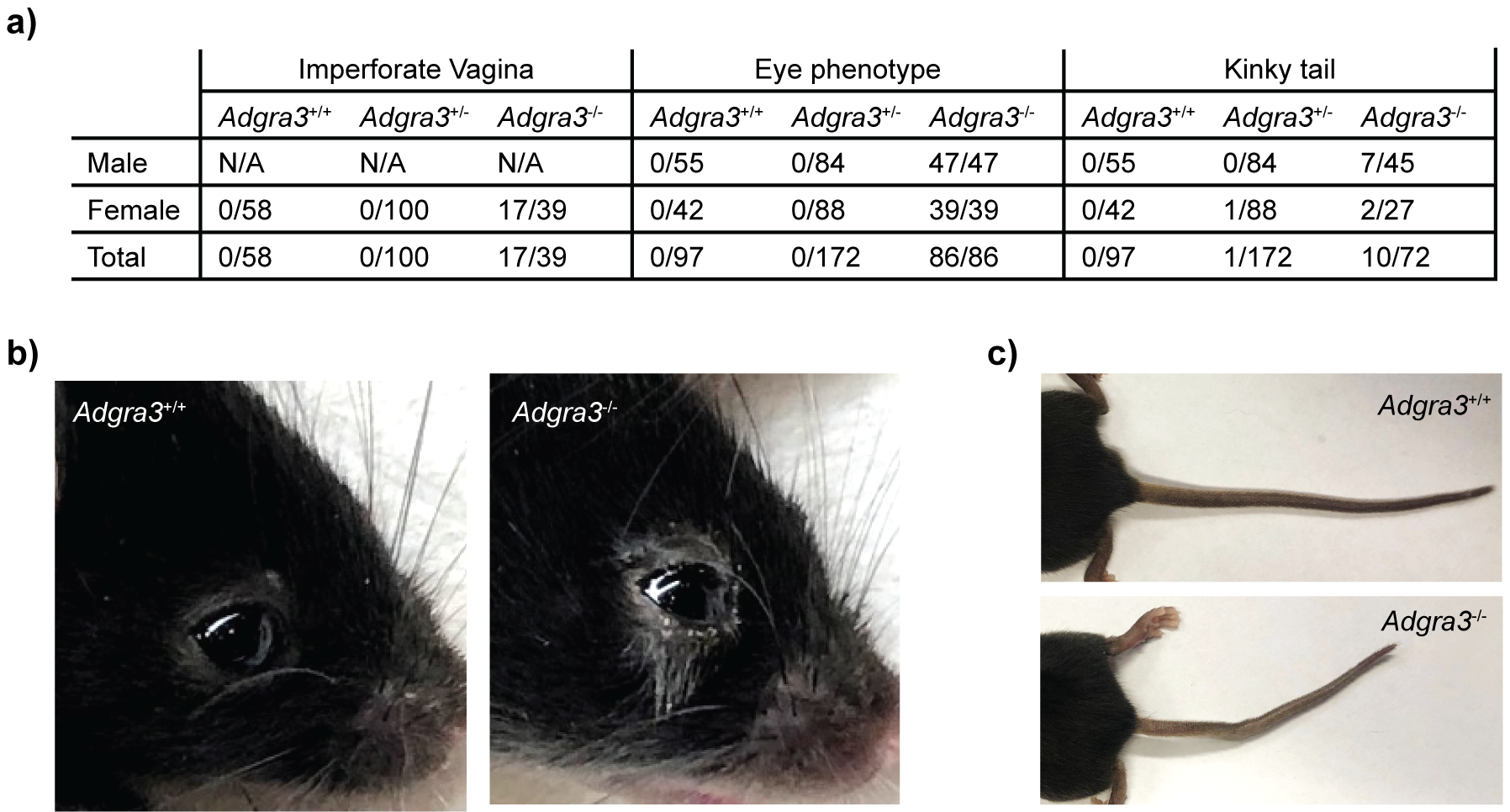

Supplement: Supplementary file 2 — Additional file 2: Figure S2. Observed phenotypes in Adgra3-/- mutant mice. (a) Incidence of phenotypic traits: imperforate vagina, eye phenotype, and kinked tail. (b) Representation of the eye phenotype observed in all Adgra3-/- mice. (c) A representative image of the kinked tail observed in 14% of Adgra3-/- mice. The kinky tail occurrence did not co-segregate with the imperforate vagina and was not further investigated. [file 12915_2024_1873_MOESM2_ESM.tif]

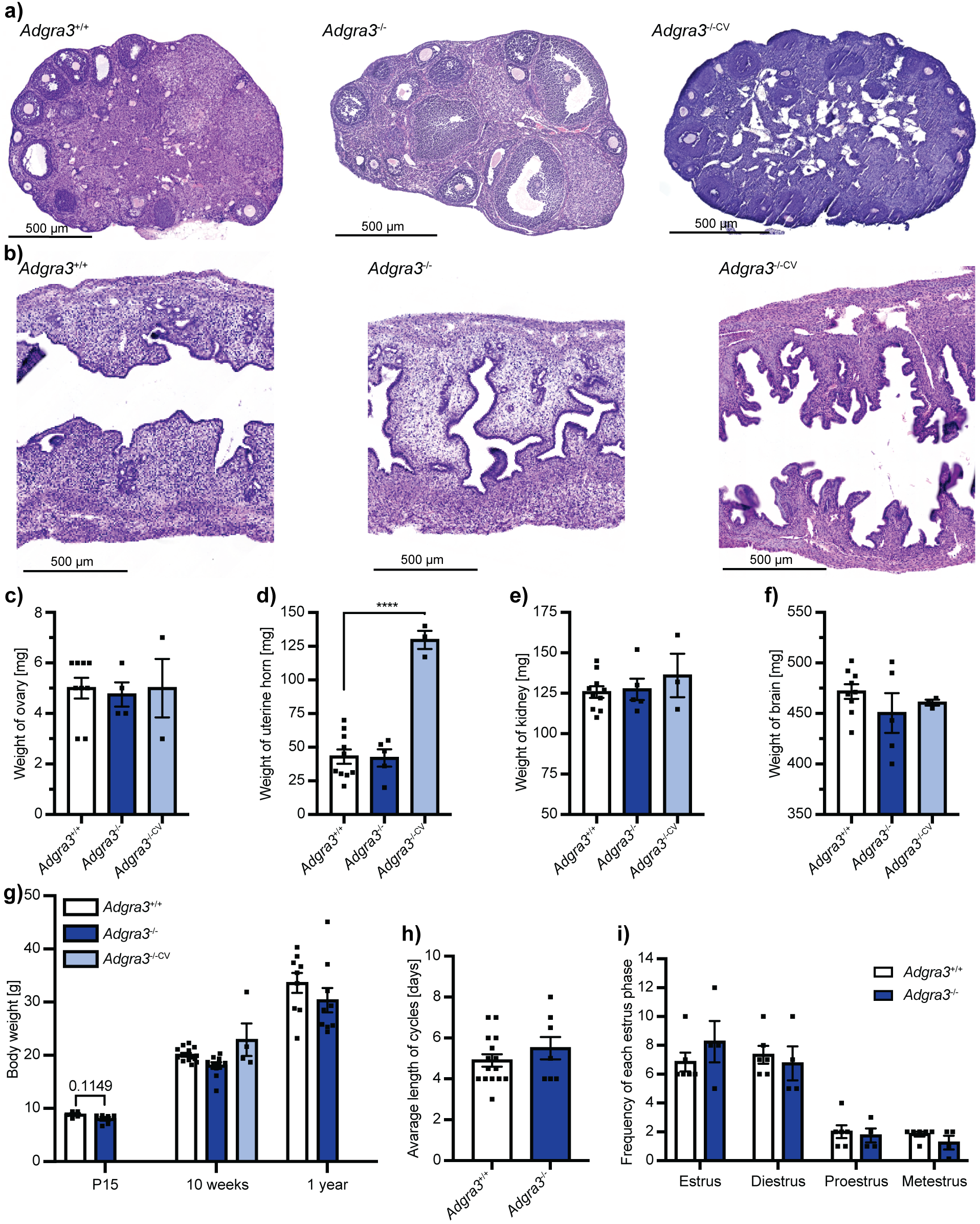

Supplement: Supplementary file 3 — Additional file 3: Figure S3. Ovarian and uterine morphology, weight of adult organs, body weight, and estrus cycle of experimental groups. (a) HE-stained whole ovary sections from Adgra3+/+, Adgra3-/-, and Adgra3-/-CV mice. (b) HE-stained sections of the middle part of the right uterine horn in Adgra3+/+, Adgra3-/-, and Adgra3-/-CV mice. The uterine morphology is disturbed in Adgra3-/-CV, possibly due to the large fluid accumulation within the uterine horns. (c-f) Weight of (c) ovary, (d) uterine horn, (e) kidney, and (f) brain collected from 8- to 10-week-old Adgra3+/+ (n = 9), Adgra3-/- (n = 5), and Adgra3-/-CV (n = 3) mice. (g) Body weight of the age subgroups used in this study. P15: Adgra3+/+ (n = 5) and Adgra3-/- (n = 6); 8- to 10 weeks: Adgra3+/+ (n = 9), Adgra3-/- (n = 5), Adgra3-/-CV (n = 3); 1 year: Adgra3+/+ (n = 9) and Adgra3-/- (n = 9). Each dot represents an individual animal. (h) The average length of each estrus cycle of Adgra3+/+ (n = 6) and Adgra3-/- (n = 4) mice with an open vagina. Each dot represents one cycle counted as the days from one proestrus to the next. (i) The frequency of estrus, diestrus, proestrus, and metestrus observed in Adgra3+/+ (n = 6) and Adgra3-/- (n = 4) females with an open vagina are presented as the number of times each phase was recorded over 17 days. Data are presented as mean ±SEM. ****p < 0.0001. [file 12915_2024_1873_MOESM3_ESM.tif]

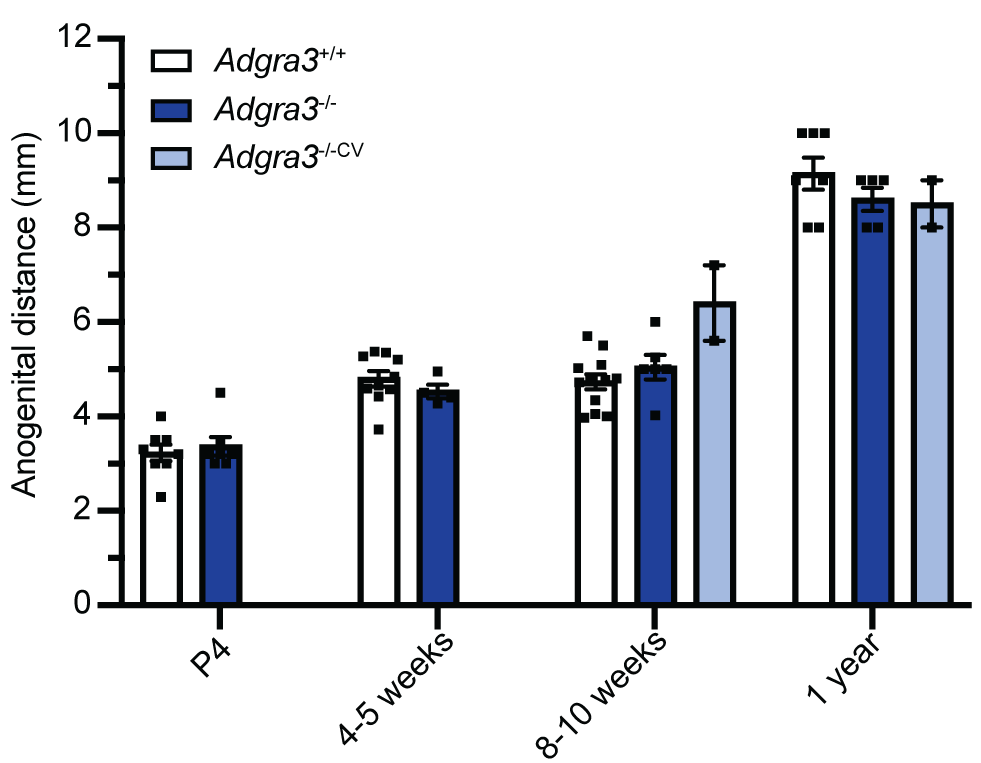

Supplement: Supplementary file 7 — Additional file 7: Figure S4. Anogenital distance in ADGRA3-deficient females compared to wild-type at postnatal day 4, 4–5 weeks, 8–10 weeks, and 1 year of age. Anogenital distance measured between the rectum and the center of the genital papilla in 4-day-old (Adgra3+/+n = 7, Adgra3-/-n = 7), 4 to 5-week-old (Adgra3+/+n = 10, Adgra3-/-n = 4), 8- to 10-week-old (Adgra3+/+n = 12, Adgra3-/-n = 6, Adgra3-/-CVn = 2), and 1-year-old females (Adgra3+/+n = 7, Adgra3-/-n = 5, Adgra3-/-CVn = 2). Each dot represents an individual animal. [file 12915_2024_1873_MOESM7_ESM.tif]
